# Spiers Memorial Lecture: Vibrations at interfaces

**DOI:** 10.1039/d6fd00055j

**Published:** 2026-05-08

**Authors:** Ellen H. G. Backus, Tobias Dickbreder, Manuel Hofmann, Clara-Magdalena Saak, Moritz Zelenka

**Affiliations:** a Institute of Physical Chemistry, Faculty of Chemistry, University of Vienna Währinger Strasse 42 Vienna 1090 Austria; b University of Vienna, Vienna Doctoral School in Chemistry (DoSChem) Währinger Strasse 42 Vienna 1090 Austria ellen.backus@univie.ac.at

## Abstract

As the transition region between bulk phases, interfaces are of crucial importance for a wide range of natural and technological fields, including heterogeneous catalysis, nucleation and growth, and wetting. As such, there is a great interest in understanding the molecular structure and properties of interfaces to develop and advance technological applications. Vibrational spectroscopy is a powerful tool to assess the molecular structure and environment. However, spectroscopic studies of interfaces can be challenging, since the interfacial region is usually small compared to the contacting bulk phases, leading to small signals and selectivity problems. In this introductory chapter to the Faraday Discussion “Vibrations at Interfaces”, we discuss selected examples to highlight how these challenges can be overcome to study interfaces using vibrational spectroscopy and which research questions can be answered.

## Abstract 2

Highlighting the state-of-the-art for time-resolved and static vibrational techniques to study interfaces in different environments.

## Graphical abstract

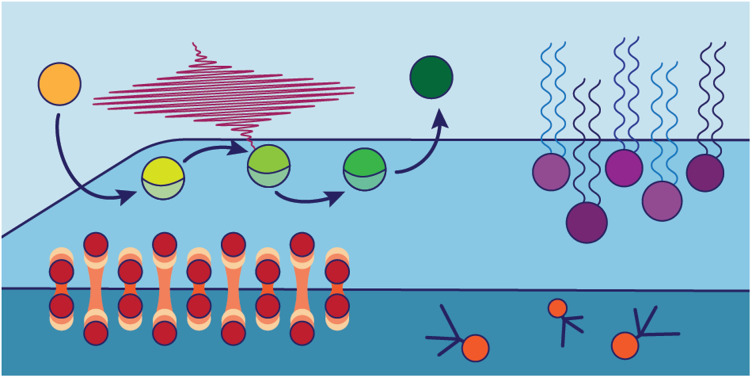

## 1 Introduction

Interfaces form at the contact face between two bulk phases, and, as such, interfaces mediate all interactions between these phases. Therefore, interfaces play a central role in diverse systems and applications with environmental, biological and technological significance. Examples for interface-specific processes range from nucleation and growth to heterogeneous catalysis or wetting phenomena. The structure and properties of the interfaces involved in these examples vary widely depending on the characteristics of the contacting bulk phases. For example, interfaces exist between phases with the same aggregate state, like liquid–liquid interfaces, and between phases with different aggregate states like solid–liquid or liquid–gas interfaces. Due to their widespread importance, there is significant interest in the molecular structure and properties of interfaces to understand interfacial processes and develop technological applications. However, the study of interfaces is also especially challenging, because the interfacial region is inherently small compared to the bulk phases, leading to generally low signal intensities and sensitivity problems.

A powerful approach to gain direct molecular-level insight into specific molecular moieties and their chemical environment is vibrational spectroscopy. The spectral range of a vibrational frequency reports on the type of molecular group: the C=O stretch vibrations, for example, are found at around 1600–1700 cm^−1^, the C–H stretch vibrations around 2800 to 3100 cm^−1^, and the O–H stretch vibrations between 3000 and 4000 cm^−1^. Furthermore, precise analysis of the vibrational frequency can additionally provide information about the molecular structure and/or environment. In particular, the C=O vibration of a peptide bond is sensitive to the secondary conformation of a protein: the vibrational frequency is different for an α-helix or a β-sheet conformation.^1^ Other examples are the O–H stretch vibration frequency being influenced by hydrogen bonding, which red-shifts the frequency of the mode upon stronger H-bonding,^2^ and the different vibrational frequency for graphene and graphite.^3^

**Fig. 1:**
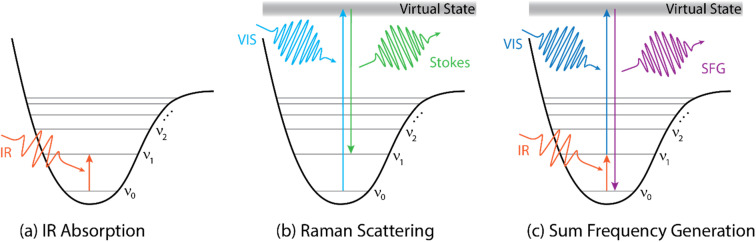
Energy diagrams describing IR absorption (a), Stokes-Raman scattering (b), and sum-frequency generation (c).

In the bulk phase, the vibrational spectrum is directly accessible by conventional transmission infrared (IR) spectroscopy, which resolves the relative loss of IR light in the sample phase across the mid IR range. This loss spectrum directly reveals the frequency-dependent absorption of IR light *via* the vibrational resonances (as sketched in Fig. 1a) – when neglecting scattering terms.

Alternatively, vibrational spectra of bulk samples can also be measured with Raman spectroscopy, which has the advantage of using visible light to irradiate the sample. The visible light is inelastically scattered by the sample. The frequency difference between the scattered and incoming light contains information about vibrations of molecular groups (Fig. 1b). As the selection rules of IR and Raman spectroscopy are different (see for example ref. 4), both methods often provide complementary information. For example, some vibrational modes might only appear in the IR or in the Raman spectrum. Moreover, Raman spectroscopy can be advantageous for samples that are highly absorbing in the IR range, as the incoming and scattered light are in the visible range and may illuminate a larger probe volume.

While IR and Raman spectroscopy are powerful techniques, neither method is interface selective. Instead, all illuminated molecules in a sample are spectrally interrogated, which is why information about surface species is often masked by the overpowering bulk signal. This is mainly due to the number of interfacial molecules being diminishingly small compared to that of the bulk.

To solve this missing interfacial selectivity while maintaining the advantages of vibrational spectroscopy, several interface-sensitive vibrational spectroscopy techniques have been developed over the years. These techniques can conceptually be separated into two families, which make use of fundamentally different strategies to access interface-specific spectral information: the first relies on signal enhancement at the interface, while the second employs strategies to suppress the bulk signal entirely.

Second order non-linear optical methods, like sum-frequency generation (SFG) spectroscopy (Fig. 1c), fall into the second category, as no SFG signal is generated in the centrosymmetric bulk medium.^5^ Interfacial signal enhancement, on the other hand, can be obtained by, *e.g.*, reflection–absorption infrared spectroscopy (RAIRS or IRRAS) or *via* methods using total internal reflection or surface plasmons.^6^

In the current chapter we aim to introduce the topic of “vibrations at interfaces” and discuss different strategies to obtain interface-sensitive vibrational spectroscopy. Due to the wide variety of available techniques, we do not aim for an exhaustive overview but will highlight selective examples illustrating the capabilities of different methods (*i.e.*, “what is the question, what is the technique”) and their application to the “analysis of complex systems” and time-resolved studies for “tracking change”. For selected examples, simulation results are briefly mentioned (“the surface *in silico*”). We have structured the manuscript around three commonly used spectroscopic techniques: infrared, Raman, and sum-frequency generation spectroscopy. Finally, we provide a short overview of recent advances addressing longstanding challenges in the field.

## 2 Infrared spectroscopy

As mentioned in the introduction, infrared spectroscopy probes molecular vibrations through wavelength-dependent absorption of IR light, providing a chemical “fingerprint” that reports on bond types, functional groups, and local environment.^7^ A vibrational mode is IR-active if it involves a change in the molecular dipole moment. As such, infrared spectroscopy is not by default surface-sensitive, but specialized methods or sample systems are needed to measure vibrations specifically at interfaces.

### 2.1 Surface-enhancement methods in internal reflection geometry

For studying solid surfaces, such as minerals or electrodes, in contact with bulk solutions, a certain amount of surface sensitivity can be obtained by measuring in attenuated total reflection (ATR) geometry. ATR uses a crystal with a high refractive index (*e.g.*, diamond, ZnSe, Ge). The IR light illuminates the interface from the crystal side at an angle of incidence with the surface normal above total internal reflection. The resulting evanescent field penetrates only a few hundred nanometres into the sample, probing the near-surface region. The effective penetration depth depends on the wavelength, incident angle, and refractive indices. ATR is now a default method for many condensed-phase measurements.^7^

To further enhance the surface sensitivity to the nanometer scale, rough metallic films deposited on the crystal, which produce surface plasmons upon excitation with p-polarized light (*i.e.*, parallel to the plane of incidence), can be used.^8^ Surface plasmons strongly enhance the local electric field at the interface, increasing the signal strength, and give the technique its name of surface-enhanced infrared absorption spectroscopy (SEIRAS). Due to specific surface selection rules, vibrational bands arising from a change in dipole moment perpendicular to the surface are enhanced over the parallel ones, resulting in information about the orientation of molecular moieties.^8^ This method has, for example, enabled the study of biological systems by attaching lipid bilayers to a SEIRA-active gold film. In this way the Heberle group^9^ could characterize a folding intermediate in the pH range between pH 7.0 and 5.3 in the insertion of the pH-low insertion peptide (pHLIP) into a solid-supported lipid bilayer.

Often the metal layer itself is the substrate of interest, as the metal forms the electrode. This principle is illustrated in the spectroelectrochemical investigation of CO_2_ to CO conversion on nanoporous structured gold deposited on a Si prism. The authors^10^ could conclude using *in situ* SEIRAS that this nanosponge gold layer promotes the linear CO intermediate over the bridging CO intermediate. Dynamics can be obtained by measuring subsequent ATR-SEIRAS spectra, resulting in a typical resolution on a timescale of seconds. Higher time resolution up to sub-picoseconds can be achieved *via* step-scan FTIR and pump–probe spectroscopy. Details can be found in the literature.^11–13^

### 2.2 Reflection–absorption infrared spectroscopy

Reflection–absorption IR spectroscopy (RAIRS or RAIS) is commonly used to study (sub)monolayer adsorption on single crystals in ultra-high vacuum (UHV) or even under high pressure. The method is also known under the names infrared reflection–absorption spectroscopy (IRRAS or IRAS) or external reflection spectroscopy (ERS) depending on the specific experimental geometry and research community. What unifies these approaches is the fundamental method: the IR light is reflected once on the surface before it enters a Fourier transform infrared spectrometer. On metal surfaces, selection rules dictate that only vibrational modes with a dipole change perpendicular to the surface can be detected,^6^ thus providing information on the orientation of the adsorbate. This method has been very powerful for identifying which species are present on the surface and to determine their environment. However, as recently reviewed, dipole–dipole coupling might make assignment of an observed band to adsorbed species difficult, a problem that might be resolved by using mixed isotopes.^14^ A very recent example by Fredersdorff *et al.*^15^ shows the sensitivity of RAIRS spectra to the environment of molecules. They use CO as a probe molecule to characterize single-atom alloys for heterogeneous catalysis. Moreover, the kinetics of surface processes, such as the dimerization kinetics of methyl pyruvate (CH_3_CCO_2_CH_3_) on Pd(111) or the activation energy for multilayer desorption, could be studied by performing a slow temperature ramp while continuously measuring RAIRS spectra.^16^

**Fig. 2:**
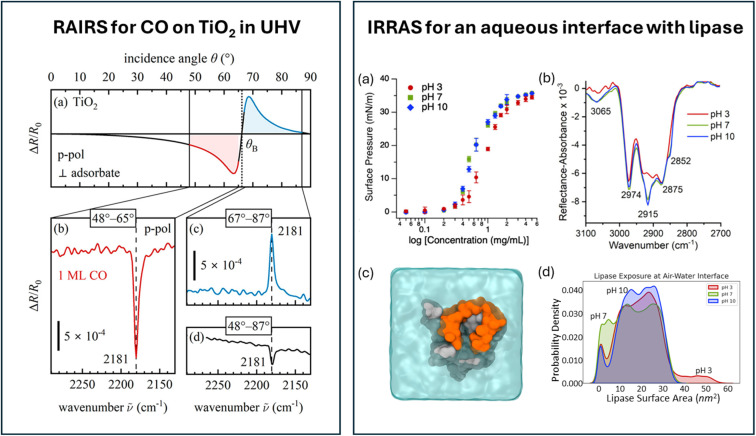
(Left) (a) Calculated angle dependence of Δ*R*/*R*_0_ for CO on TiO_2_(110); (b–d) experimental RAIRS spectrum for 1 ML of CO on TiO_2_(110) at different angle-of-incidence ranges. Graphic taken from ref. 17 under the CC-BY 4.0 license agreement. (Right) (a) Surface pressure as a function of concentration for *Burkholderia cepacia* lipase (BCL) in aqueous solutions of different pH; (b) IRRAS spectra of BCL at different pH at the air–water interface in 0.4 M NaCl; (c) top–down snapshot of an MD simulation of BCL at the air–water interface; (d) distribution of the exposed lipase surface area at the air–water interface. Graphic adapted from ref. 18 under the CC-BY 4.0 license agreement.

In contrast to the large number of RAIRS studies on adsorbates on metal surfaces, semiconductor surfaces are far less commonly studied using this method. One reason is the significantly weaker signal. In 2024, Rath *et al.* described an infrared reflection setup designed specifically to measure non-metals.^17^ In contrast to metals, vibrational modes in any orientation can be measured: s-polarized light probes molecules parallel to the surface, while p-polarized light probes both parallel and perpendicular modes. High sensitivity is achieved by optimizing the light throughput and the incident angles, as is clear from panel a in Fig. 2 (left), showing the calculated angle-dependent absorbance of CO on TiO_2_(110). In contrast to RAIRS from metals, where only positive Δ*R/R*_0_ signals are observed, the signal measured with p-polarized light on semiconductors changes sign at the Brewster angle, annotated with *θ*_B_ in the graphic. Great care must therefore be taken to finetune the incident angle, as seen in Fig. 2 (left panel, b to d), which depicts the stretch vibration of CO (at 2181 cm^−1^) at monolayer coverage, obtained in about 5 minutes of acquisition time (1000 scans). The signal to noise ratio is naturally reduced if the positive and negative spectral components are summed up upon signal collection over the wide angular range of 48° to 87°. Moreover, in about 20 minutes (4000 repetitions), a decent spectrum of a 0.1 monolayer of CO using p-polarized light or a spectrum of a monolayer of D_2_O in s-polarization could be measured.

Infrared reflection methods have also been applied to aqueous interfaces, with the more common name for the method in this community being IRRAS. Unfortunately, the signal intensity in these systems remains very weak, as no enhancement effects are present. An example is the pH-dependent study of *Burkholderia cepacia* lipase, a model enzyme found in marine environments, at the water–air interface, shown in the right panel of Fig. 2. The authors focussed on the C–H vibrations, as this region is free from water vapour and has a reasonable signal intensity.^18^Fig. 2 right, panel b, shows vibrations in the range of 2700 to 3000 cm^−1^, assigned to symmetric and asymmetric stretches of methyl and methylene groups. The vibrational mode at 3065 cm^−1^ is attributed to the C–H stretch mode of the phenyl groups. Upon increasing the pH, the bands at 2915 and 3065 cm^−1^ increase in intensity. Combined with surface pressure results (Fig. 2 right, panel a), the authors conclude that the lipase has higher surface propensity at higher pH. All-atom molecular dynamics simulations show that the surface-exposed area of the lipase at pH 3 is significantly larger than at pH 7 and 10, which is clear from the distribution of the lipase surface area depicted in panel d of Fig. 2 (right). The authors explain the larger surface area at pH 3 *via* an extended open-lid conformation.

### 2.3 Diffuse reflectance infrared Fourier transform spectroscopy

Another strategy to make IR spectroscopy more surface-specific is to significantly increase the surface area by investigating powdered samples. This strategy is especially suitable for catalytic studies, which commonly investigate powders instead of flat samples to increase the reactive surface area. An infrared-based method highly suitable for measuring powders is diffuse reflectance infrared Fourier transform spectroscopy (DRIFTS). In this method,^19^ the diffuse reflected light is collected by an ellipsoidal mirror and subsequently detected in an FTIR spectrometer.

Despite the large surface area, it is often very difficult to measure reaction intermediates, as they are only present in small amounts. Additionally, their signal might be masked by backgrounds from the catalyst or by spectator species. One way to obtain sensitivity for intermediates is by modulation excitation spectroscopy (MES) in combination with phase-sensitive detection (PSD) analysis. In this method, a periodic concentration perturbation is introduced in the reaction system. Details of the method and the analysis can be found in ref. 20.

**Fig. 3:**
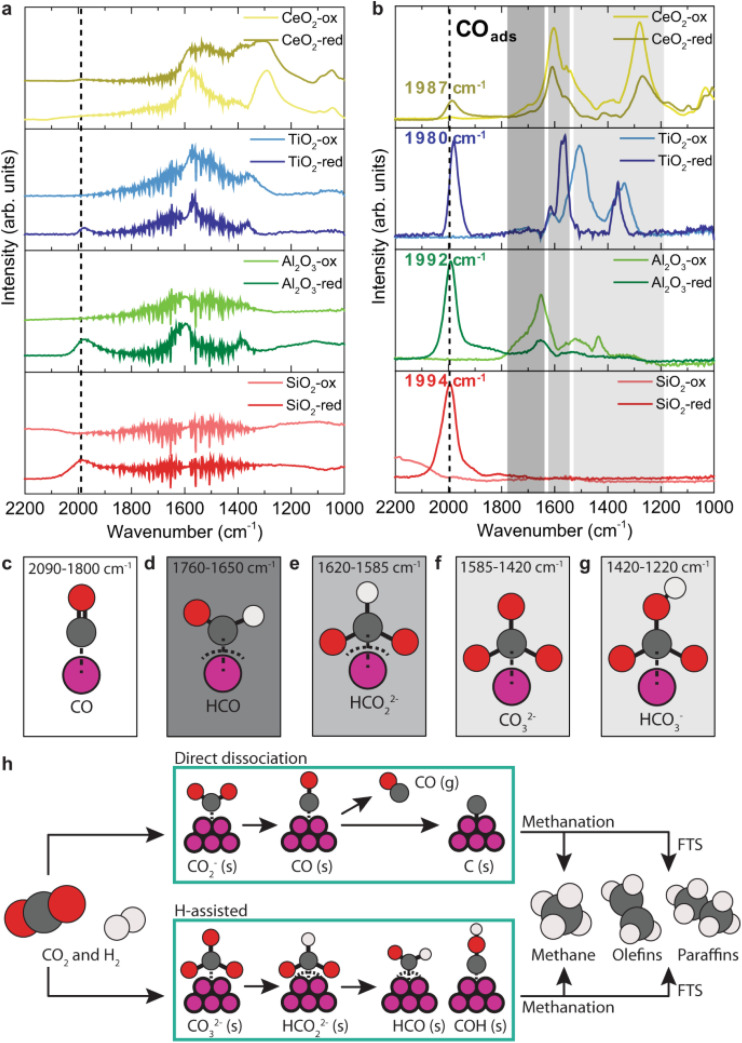
(a) Averaged time-resolved DRIFT spectra during CO_2_ hydrogenation with cobalt-based catalysts. (b) Phase-resolved amplitude spectra of CoO (suffix: -ox) and metallic Co (suffix: -red) supported catalysts (*T* = 250 °C, *P* = 1 bar, H_2_/CO_2_ = 3). (c–g) Characteristic vibrational frequencies and their adsorbed species, labelled in the graphics. (h) Schematics of the simplified reaction pathways of CO_2_ hydrogenation to hydrocarbons on the cobalt-based catalysts. Graphic taken from ref. 21 under the CC-BY 4.0 license agreement.

As an illustrative example of the method, we discuss here the CO_2_ hydrogenation reaction on cobalt and cobalt oxide nanoparticles.^21^Fig. 3a shows the time-averaged DRIFT spectra for the nanoparticles on different supports upon exposure to a mixture of CO_2_ and H_2_. The spectra show several vibrational features between 1200 and 2200 cm^−1^, the details of which depend on the support and on the nanoparticles being CoO or Co. The phase-resolved amplitude spectra in Fig. 3b show much more pronounced peaks, especially at 2000 cm^−1^. This band is assigned to adsorbed CO. The precise frequency reports on the binding strength between the metal atom and the CO: a stronger binding results in a weaker C=O bond and thus a lower vibrational frequency. The strongest CO binding is correlated with the highest activity.^21^ Additionally, Fig. 3b shows pronounced differences between reduced and oxidized samples. In particular, the band at 2000 cm^−1^ is absent for the oxidized surface, while other bands at lower frequencies, assigned to different types of CO-containing adsorbed species (Fig. 3c–g), are more pronounced. As different species are observed in the spectrum for the two states of the catalyst, different reaction mechanisms are concluded, as schematically depicted in Fig. 3h. For the metallic Co case, C=O is an intermediate, indicating that the hydrogenation follows a direct dissociation mechanism. In the case of CoO, the reaction path follows an H-assisted mechanism.

### 2.4 Solvent-shell spectroscopy

The solvation shell and its properties are of particular importance in biochemistry, where solvent interaction and incorporation play an important role in, *e.g.*, protein structure (proteins in saline solution^22,23^) or cellular processes. Besides supramolecular chemistry, the solvation shell also plays a decisive role in the reactivity, aggregation, and dynamics of solvated species.^24–26^

**Fig. 4:**
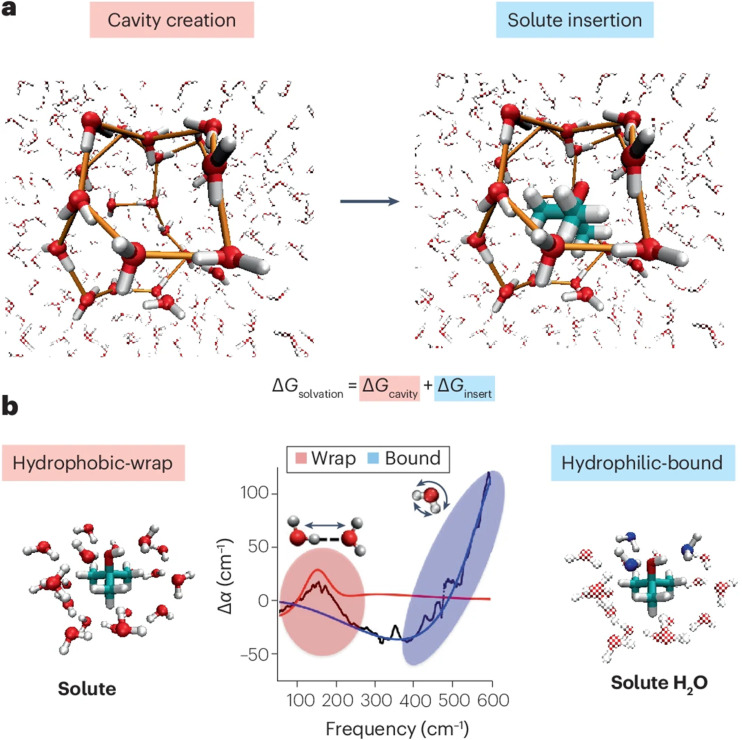
(a) Concept of cavity creation and solute insertion determining the solvation energy. (b) Illustration of the spectral observation at around 150 cm^−1^ and above 300 cm^−1^ for the cavity, *i.e.*, hydrophobic wrap, and the hydrophilic bonding. Reproduced from S. Pezzotti, W. Chen, F. Novelli, X. Yu, C. Hoberg and M. Havenith, Terahertz calorimetry spotlights the role of water in biological processes, *Nat. Rev. Chem.*, 2025, **9**, 481, with permission from Springer Nature.^28^

The solvation shell is sometimes defined as a molecular interface, especially for macromolecules or when the solute has a (partial) hydrophobic character. This characterization can in part be rationalized in relation to the cavity formation process, which is an inherent step of the solvation process – which we will briefly describe using water as the exemplary solvent. When a solvation shell is formed around any solute, the reorientation of the solvent can be described as a two-step process,^27,28^ which is sketched in Fig. 4a for *tert*-butyl alcohol with water as the solvent. Initially, a cavity is formed. In the second step, the solute is inserted and the specific interaction of the solute with the solvent molecules in its direct vicinity can lead to further reorientation, determined by hydrophilic interaction. These two contributions have respectively been described as the predominantly hydrophobic and hydrophilic part of the solvation shell, and lead to a characteristic perturbation of the intermolecular solvent network. Accessing the two terms experimentally would allow for the underlying thermodynamics of the solvation process to be determined. This has been challenging, since intramolecular modes of the solvent are often sensitive to both aspects of the solvation process, thus making their disentanglement inaccessible.^27,28^

One approach, which has recently emerged, makes use of the water response in the terahertz (THz) region, which predominantly reports on intermolecular vibrational modes, and as such is inherently sensitive to the local hydrogen-bond network. The unique capability of this spectral window has been demonstrated in recent work from the Havenith group,^28^ who have shown that both contributions to the solvation process can be individually and quantitatively characterized in the THz region of around 100 to 600 cm^−1^. The authors show that water that wraps around the solute (hydrophobic-wrap) and water that is hydrophilic-bound to the headgroup of the solute spectrally contribute to different regions of the THz spectrum (see Fig. 4b). This is the case because the two aspects are more strongly correlated with spectral changes of two different vibrational modes: the hydrophobic-wrap is correlated with the intermolecular hydrogen-bond stretch mode, and the *hydrophilic-bound* with the librational mode (see Fig. 4b). Extracting these contributions allows for the Gibbs free energy of cavitation and solute insertion to be determined, making the fundamental thermodynamics of solvation directly accessible through the experimental measurement and giving the technique its name of terahertz calorimetry.

Another approach to study the solvation shell is by isolating its spectroscopic features in the conventional, mid-IR, vibrational range using IR^22,23,29^ or Raman^30–32^ spectroscopy. In aqueous solutions, the hydrogen-bonded OH stretch band is often targeted, as it also shows a strong correlation between the local hydrogen-bond environment and the vibrational frequency.^2^ In the most straightforward approach, this method allows the bulk solvent contribution to be removed from the spectrum of a solution, revealing the spectral component of the solute and its solvation shell. This residual spectral contribution is referred to as the solute-correlated spectrum. This approach is especially appealing for low-concentration solutions or in the case where the solvent has a particularly high absorbance, (in part) masking the spectral contribution of the solute.

Removal of the spectral component of the bulk solvent can be performed *via* multivariate curve resolution (MCR),^30^ self-modeling curve resolution (SMCR),^33^ or by hand,^29^ ensuring non-negative bands across the entire spectral range – which differentiated this approach from regular difference spectroscopy. An example where this approach was applied in IR spectroscopy is that of *tert*-butyl alcohol in a mixed solvent of water and propanol.^29^ To investigate how the mixed solvation shell around *tert*-butyl alcohol changes across the compositional range, the fraction of propanol is varied systematically from 0 to 1. While the total OH stretch band of the solution varies smoothly across the studied range, the solute-correlated spectra reveal a non-linear change in the OH stretch band. These spectra show that the water content of the solvation decreases even at relatively low fractions of propanol in the solution, leading to an increasingly hydrophobic local environment. These solvation-shell spectra are put into context by MD simulations of the local solvation environment, which reveal a highly structured solvation environment. It is especially notable that the mixed solvent forms shells of alternating hydrophilic–hydrophobic character around the *tert*-butyl alcohol solute.

In analogy, Raman spectroscopy in combination with spectral decomposition can also be used to study the local hydration environment.^30^ Initially, the method was used to detect water in local hydrophobic environments of molecular solvation shells, having sufficient sensitivity to observe even dangling hydrogen bonds,^31^ indicative of a frustrated hydrogen-bond network around hydrophobic moieties. The authors were also able to observe a shift in the free-OH frequency when the free OH formed a weak hydrogen bond with the aromatic system of benzyl alcohol. Enhancing the complexity, the hydration of larger, supramolecular structures, such as micelles, can similarly be studied.^33^

## 3 Raman spectroscopy

Raman spectroscopy uses inelastic light scattering to access molecular vibrational information. When a monochromatic beam hits the sample, a small fraction of the light is inelastically scattered with energies shifted relative to the incident light. If the molecule gains vibrational energy, the scattered photon is lower in energy (Stokes), and if the molecule loses vibrational energy, the scattered photon is higher in energy (anti-Stokes). This frequency shift provides a vibrational “fingerprint” complementary to infrared absorption.^34^

Raman scattering is intrinsically weak, so instruments use narrow-band lasers to deliver high spectral brightness and stability. Because the light source is usually in the visible or near-IR, standard microscope optics, coverslips, and immersion media can be effectively used. Modern Raman instruments commonly employ confocal microscopy to acquire spectra with micrometer-scale spatial resolution. A high-numerical-aperture objective focuses the laser beam in the best case to a diffraction-limited spot. This increases the local irradiance by orders of magnitude and thereby boosts sensitivity, since the Raman signal scales linearly with the intensity of the incident light beam. Adding an image-plane pinhole yields confocal Raman microscopy, which rejects out-of-focus light and enables depth-resolved, high-contrast chemical imaging.^35^

### 3.1 Surface- and tip-enhanced Raman spectroscopy

Due to the relative inefficiency of the Raman process, obtaining surface specificity is especially challenging. In analogy to the infrared experiments described in Section 2.1, Raman signals can be enhanced by an internal reflection geometry in combination with a thin metal film, resulting in surface plasmons. In this way, enhancement factors of roughly 10–100 times have been reported.^36^ The foundation for surface enhancement in Raman spectroscopy dates to the mid-1970s, when spectra of pyridine at an electrochemically roughened silver electrode were measured; this enhanced signal was first reported at a Faraday Discussion in 1973 in Oxford.^37,38^ For the subsequent historical development, we refer to a recent review.^36^ Enhancement factors of over 10^6^ are observed if localized surface plasmons are involved, obtained by using nanostructured materials.

**Fig. 5:**
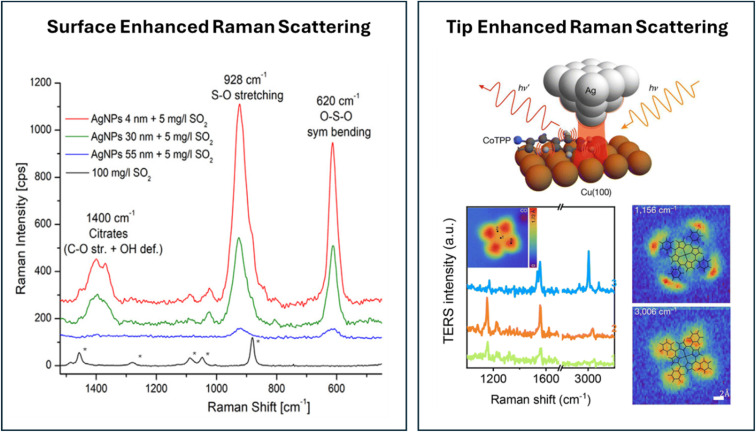
(Left) Raman spectra for solutions of SO_2_ in EtOH/H_2_O with and without the presence of silver nanoparticles. Reprinted from L. Mandrile, I. Cagnasso, L. Berta, A. M. Giovannozzi, M. Petrozziello, F. Pellegrino, A. Asproudi, F. Durbiano and A. M. Rossi, Direct quantification of sulfur dioxide in wine by Surface Enhanced Raman Spectroscopy, *Food Chemistry*, 2020, **326**, 127009, Copyright (2020), with permission from Elsevier.^39^ (Right) Principle of a TERS experiment, illustrated for a Co(ii)-tetraphenylporphyrin (CoTPP) molecule immobilized on Cu(100) under UHV at 6 K. Reproduced from ref. 36 with permission from the Royal Society of Chemistry.

An illustrative example of the potential of surface-enhanced Raman spectroscopy (SERS) is depicted in Fig. 5 (left panel). In these experiments,^39^ Ag nanoparticles of different sizes are added to a solution to monitor the presence of SO_2_. In a Langmuir-like adsorption–desorption process, the SO_2_ molecules adsorb on the Ag nanoparticles. For a 100 mg L^−1^ solution of SO_2_ in EtOH/H_2_O, no Raman signal of the SO_2_ is obtained; the spectra show only bands related to EtOH. However, upon adding Ag nanoparticles, very clear signals for SO_2_ are observed in the spectrum, although the SO_2_ concentration is 20 times lower. Moreover, the signal intensity depends on the particle size, which is most likely due to the greater specific surface area for small nanoparticles. The authors subsequently show that the addition of nanoparticles in SERS could be used for quantifying the SO_2_ concentration in wine. Nowadays, analytical sensing is one of the prominent directions in which SERS is finding application and method development. Another direction is so-called electrochemical SERS, which is not only used for the study of electrocatalysis, but also in analytical sensors by, for example, enhancing binding through changing the potential. More details about the principles and application of this electrochemical version of the method can be found in ref. 40–42.

To probe atomically flat surfaces with Raman, the SERS method is inapplicable. This led to the development of tip-enhanced Raman spectroscopy (TERS). In this method, a tip is used to boost the local field in the tip-sample junction, as depicted in the top figure of the right panel of Fig. 5. The potential of the method is nicely illustrated by the TERS intensity spectra as a function of the Raman shift, shown in the bottom part of the right-hand panel of Fig. 5. By moving the tip over the surface, spatially resolved Raman spectra of a Co(ii)-tetraphenylporphyrin molecule immobilized on Cu(100) under UHV at 6 K are obtained.^43^ The spectra measured at different local points and the 2D maps of the signal at 1156 cm^−1^ (phenyl bending) and 3006 cm^−1^ (C–H stretch) demonstrate the spatial resolution and the sensitivity of the method.

## 4 Sum-frequency generation spectroscopy

Sum-frequency generation (SFG) spectroscopy is a powerful technique to probe molecular vibrations at interfaces. The basic principle of an SFG experiment is spatially and temporally overlapping two high-intensity laser pulses at the sample surface to generate light at the sum frequency of both incoming lights:*ω*_Vis_ + *ω*_IR_ = *ω*_SFG_

In vibrational SFG, the *ω*_Vis_ beam is typically fixed in the visible or near-IR range and the other beam *ω*_IR_ is in the mid-IR range with a tunable frequency. The SFG intensity is recorded as a function of *ω*_SFG_. If *ω*_IR_ is in resonance with a molecular vibration, the SFG signal gets resonantly enhanced, which is shown as peaks in the spectrum.

Since SFG is a second-order non-linear process, the SFG response is only nonzero in media lacking inversion symmetry, making it inherently surface-specific.^44^ This characteristic makes it a powerful and widely employed method in studying interfacial molecular structure and dynamics in different systems, with applications spanning from biological and soft matter to solid interfaces. Using different polarization combinations of the incoming and outcoming light beams provides orientational information.^45^

Another way to obtain orientational information is performing phase-resolved (or heterodyne-detected) SFG spectroscopy. In the conventional type of SFG spectroscopy, the SFG intensity is measured and is proportional to the square modulus of the complex second-order susceptibility *χ*^(2)^, which encodes the molecular response. In this type of experiment, the complex phase of *χ*^(2)^ and all orientational information contained therein are inherently lost. However, upon interference with a so-called local oscillator signal, the complex value of *χ*^(2)^ can be directly extracted. In a simplified picture, the sign of the imaginary part of *χ*^(2)^ provides information on whether molecular groups are facing towards or away from the surface, as nicely illustrated in one of the first broadband phase-resolved SFG papers for water underneath a positively and negatively charged surfactant.^46^ The Im *χ*^(2)^ showed a clear opposite sign for water in the two cases.

Moreover, as pulsed femtosecond lasers are increasingly used to generate the illuminating beams, time-resolved experiments in a pump–probe matter can be performed with sub-picosecond resolution. In this case, the probe consists of the SFG probe pair, and the preceding pump pulse can be selected to induce a range of different processes.

### 4.1 Solid–liquid interfaces

Solid–liquid interfaces that have been investigated with SFG span from single-crystalline metal and oxide surfaces to carbon-based materials. In these systems, atomic-scale chemical heterogeneity and long-range electrostatics introduce distinct challenges for data analysis and interpretation. Metal-oxide interfaces represent a particularly interesting class of systems where SFG uncovers how surface hydroxylation, charging and specific adsorption shape interfacial water structure and reactivity. Studies on oxides such as Al_2_O_3_ have shown how pH-dependent surface charging modulates interfacial water orientation and adsorption behavior. Using phase-resolved SFG, it has been shown that on Al_2_O_3_, pH-dependent surface charging *via* (de-)protonation of surface hydroxyl groups leads to a pH-dependent flip in the net orientation of interfacial water across the point of zero charge.^47,48^ Moreover, combined experimental and theoretical work obtained valuable information about, for example, (i) the presence of hydrophobic sides on a macroscopic hydrophilic silica surface in contact with water,^49^ (ii) the presence of an unconventional five-coordinated silicon species upon deprotonation of a silica surface in contact with aqueous solution,^50^ and (iii) the surface charge on CaF_2_ in contact with water at low pH originating from the dissolution of fluoride ions rather than from adsorption of protons to the surface.^51^ For further information, the reader is directed to recent reviews about SFG spectroscopy on mineral–water interfaces.^52–54^

Another important aspect of SFG at metal and metal-oxide/water interfaces is that the spectra can be strongly shaped by the nonresonant (NR) electronic response of the solid and by the frequency-dependent Fresnel factors that modulate the local fields at the interface. As a result, apparent spectral structure may be dominated by the frequency dependence of the Fresnel factors rather than by true interfacial vibrational resonances, especially for broad spectral features like the hydrogen-bonded O–H stretch vibration in contact with metal films probed in internal reflection geometry.^55^

**Fig. 6:**
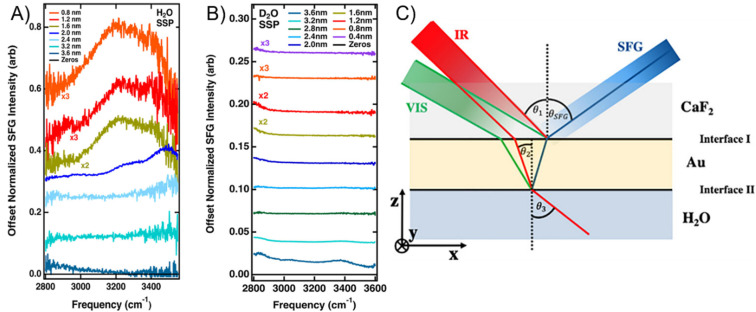
SFG spectra from the CaF_2_/Au/H_2_O system in (A) and the CaF_2_/Au/D_2_O system in (B). The spectra were taken with different thicknesses of the gold film in an ssp polarization combination. In (A), it is clearly shown that H_2_O becomes detectable from a thickness of around 1.6 nm and thinner, while the system in (B) displays a flat response, except for the 3.6 nm film. The experimental scheme used for the measurements is displayed in (C). Graphics adapted from ref. 56 under the CC-BY 4.0 license agreement.

However, for a three-layer CaF_2_/Au/H_2_O system, it has been shown that resonant interfacial water signals can be recovered when the Au layer is sufficiently thin and the polarization combination of the incident and measured beams is chosen carefully.^56^ Resonant water features on CaF_2_/Au/H_2_O can be observed in an ssp polarization combination (s-polarized SFG light, s-polarized visible light, p-polarized IR light) for Au films of around 2.0 nm and become clear at ≤1.6 nm, whereas ppp does not show these resonant features under the same conditions. Fresnel-factor simulations show that, for ssp, the enhancement factors retain a similar shape across thicknesses with a rising edge on the blue side, whereas for ppp they contain a significant spectral shape and develop a feature near 3400 cm^−1^ that becomes pronounced for thicker Au, which can mask resonances. Assignment to interfacial H_2_O is supported by D_2_O controls. In the OH region, CaF_2_/Au/D_2_O spectra are flat for ≤1.6 nm Au, with only slight features emerging at 3.2–3.6 nm (Fig. 6B). Together, these results demonstrate that recovering genuine interfacial vibrational responses at such interfaces requires explicit Fresnel-factor modelling and careful polarization/geometry choices to separate nonresonant backgrounds from resonant signals.

A careful consideration of resonant and nonresonant contributions to the SFG spectrum becomes even more important when external potentials are applied in electrochemical SFG spectroscopy. Electrode charging alters the interfacial electric field and reorganizes both the interfacial water and possible adsorbates. The measured spectrum remains a sum of resonant vibrational responses and a large nonresonant electronic background from conductive films that interferes with them.

At CaF_2_-supported graphene electrodes, heterodyne-detected SFG was used to directly read out how interfacial water responds to applied potential, showing a substrate-driven, pH-coupled pseudocapacitive process. During the hydrogen evolution reaction, water dissociation between graphene and CaF_2_ increases the local pH and charges/discharges the CaF_2_ substrate. The potential-dependent Im *χ*^(2)^ spectra match the pH-dependent spectra, showing that the dominant spectral changes arise from charging of the supporting substrate and not the graphene layer.^57^

Extending the approach to SiO_2_-supported graphene shows that the potential-induced change in the local pH is essentially identical to the CaF_2_ case. This indicates that the pH created by the electrified graphene is substrate-independent. The interfacial field sensed by water is again dominated by the charging of the substrate. This dominance reflects the substrate-dependent charging set by its isoelectric point.^58^ These studies highlight the ability of electrochemical SFG to disentangle interfacial charging and species under bias, enabling clear mechanistic insights even when substrate effects and coherent backgrounds complicate the spectra.

### 4.2 Soft-matter interfaces

Beyond its application to solid/liquid interfaces, SFG spectroscopy is also widely used to study soft matter and biological interfaces. The investigated soft-matter interfaces encompass systems such as polymer brushes,^59^ surfactant assemblies,^60^ hydrogels^61^ and supported bilayers,^62^ widely used as biological model systems. These interfaces share many common challenges of spectral congestion and dynamic restructuring.

In terms of biological systems, hierarchically organized assemblies of lipids, proteins, and water have been investigated.^63^ The heterogeneity of these systems spans from nanometers to micrometers, making them some of the most complex systems studied with SFG spectroscopy. SFG has become a central technique for interrogating these complex interfaces because it provides surface-specific orientation and conformation-sensitive vibrational information under aqueous *in situ* conditions.

Foundational work established SFG as a tool for studying aqueous interfaces and amphiphile monolayers at the air–water interface, linking spectral features to chain conformation/orientation and interfacial water structure, demonstrating how molecular ordering at soft interfaces can be resolved spectroscopically.^64^ Building on these principles, SFG spectroscopy has been applied to substrate-supported lipid bilayers and membranes to quantify phase behavior and peptide/protein–lipid interactions without exogenous labels, leveraging real-time, *in situ* measurements.^63^

One noteworthy example is the use of the amide (I, II, III) bands to access the protein secondary structure and orientation at interfaces.^65^ The amide I band (≈1600–1700 cm^−1^), dominated by the C=O stretch vibration, reports on secondary structure but exhibits strong spectral overlap between α-helical and random-coil signatures, complicating unambiguous assignment. Ye *et al.*^66^ showed that combining spectral information of the amide I (≈1600–1700 cm^−1^) and amide III (≈1200–1350 cm^−1^) bands enables more reliable secondary-structure determination and orientation analysis. Because the amide III response is typically much weaker than the amide I in SFG, technical advances are often required to achieve robust measurements. As reviewed by Hosseinpour *et al.*,^67^ near-total internal reflection geometries are one appealing approach to increase signal intensity in this spectral range.

Strategies for robust evaluation of data and against over-interpretation are especially important, as parameter non-uniqueness in resonant SFG fitting and ambiguity from nonresonant backgrounds are recognized challenges that can bias the obtained spectral information.^68,69^ One approach to overcome this problem is the introduction of heterodyne-detected SFG, where the determination of the relative phase helps to separate resonant from nonresonant contributions and resolves sign ambiguities in molecular orientation.

### 4.3 Chiral SFG

Chiral SFG works with a combination of polarisations that make the resulting emitted signal sensitive to the chirality of, *e.g.*, biological macromolecules,^70,71^ such as proteins or DNA, or the chiral assembly of achiral molecules. The latter aspect makes chiral SFG particularly powerful in the study of hydration shells in biochemical systems.

In the case of DNA, the water that constitutes the first hydration shell can populate the minor groove or the major groove, or be packed around the backbone of double stranded (ds) DNA. Perets *et al.*^70^ have recently shown that the properties of the first hydration shell can be studied in detail *via* a combined approach of experimental chiral SFG and MD simulations. The reported chiral SFG in the region of 2960–3800 cm^−1^ shows vibrational bands that can in principle be attributed to the CH and NH bands of the dsDNA, as well as the OH of water that hydrates the double strand. Since water itself is achiral, any water OH contribution to the chiral SFG spectrum would indicate that chirality has been imprinted from the chiral DNA strand onto its hydration environment. The authors can demonstrate that such a water contribution exists in their data by isotopic substitution (H_2_^18^O), which shifts only the OH band by around 12 cm^−1^.

**Fig. 7:**
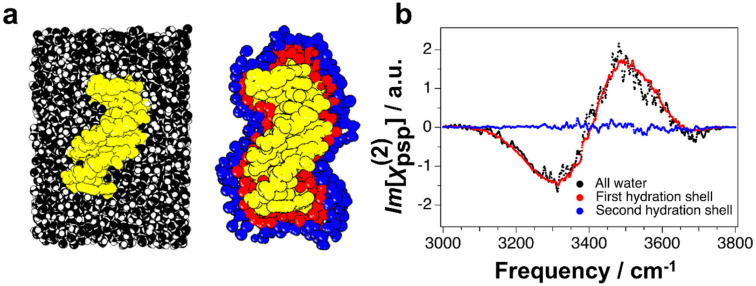
MD simulations of dsDNA (yellow) in water. (a) View of the simulation box including all water molecules in black (left) and the water molecules that constitute the first (red) and second (blue) solvation shell (right). (b) Predicted chiral SFG response of all water molecules (black) and from the first (red) and second (blue) solvation shell of water around dsDNA. Vibrational modes from the dsDNA are not calculated here. Reprinted from E. A. Perets, D. Konstantinovsky, T. Santiago, P. E. Videla, M. Tremblay, L. Velarde, V. S. Batista, S. Hammes-Schiffer and E. C. Y. Yan, Beyond the “spine of hydration”: Chiral SFG spectroscopy detects DNA first hydration shell and base pair structures, *J. Chem. Phys.*, 2024, **161** (9), 095104, with the permission of AIP Publishing.^70^

To investigate how far this chiral structuring of the solvation environment reaches into the bulk, the authors performed MD simulations of dsDNA in water, the geometry of which is shown in Fig. 7a. The authors then predicted the chiral SFG response of the total water population, as well as that of the first and second hydration shell (see Fig. 7a). The resulting predicted SFG spectrum of all water molecules in the ensemble shows the expected dispersive line shape across the OH stretch region (see Fig. 7b). When comparing the total water spectrum with the decomposition of the spectral contributions from the first and second shell, it becomes immediately apparent that the chiral response has already vanished in the second solvation shell. This is in stark contrast to the dipolar orientation of water induced by the electric field of the negatively charged backbone of the same dsDNA, which affects water molecules far beyond the initial hydration shells. These results show that while dsDNA can imprint its chiral character on the hydrating water molecules, the resulting chirality of the water ensemble is lost after the first solvation shell.

### 4.4 Time-resolved sum-frequency generation spectroscopy

Interfaces are often not static, but dynamic. For example, solids can dissolve in contact with water,^72,73^ biomolecules change their structure,^74^ or light can induce reactions.^75,76^ To track these changes, the interface needs to be monitored on the timescale of the dynamics taking place. Interfacial processes surrounded by bulk liquids and/or solids taking place on a seconds to minutes timescale can be easily followed by measuring subsequent SFG spectra in time,^77^ as a typical SFG spectrum is acquired on a minute timescale, which can even be brought down to seconds^78^ by using a total internal reflection geometry.

In contrast, to study molecular rearrangement and diffusion, energy dissipation dynamics, or molecular reactions on timescales that capture the relevant molecular processes, sub-picosecond time resolution is needed. Over the past decades, several groups used time-resolved SFG spectroscopy to study energy relaxation with a focus on water in different environments,^79–81^ but reactions on the sub-picosecond timescale at bulk interfaces are rarely studied. This is because following reactions on a sub-picosecond timescale is highly challenging due to small signals and the short timescales. Typical experiments are performed with pump–probe spectroscopy, where the pump light induces the reaction and an SFG probe pair monitors the reactions at well-defined timescales after the pump pulse. With this concept, the method is limited to studying photoreactions. Upon transferring concepts from time-resolved bulk spectroscopy, in principle, temperature-jump and pH-jump reactions might be possible as well. The use of a temperature jump was recently applied for studying the dynamics of the electric double layer at the water–air interface, which was positively charged because of the interfacial presence of protons.^82^

It is important to study interfacial reaction mechanisms, as they can be markedly different from bulk reactions, as illustrated by the photodissociation of phenol into a phenoxy radical, and a hydrated proton and electron.^76^ In the bulk, this reaction takes place on a timescale of roughly 5 ns, while the reaction proceeds in less than 0.1 ps at the air/water interface. The surface thus speeds up the reaction by roughly four orders of magnitude, probably due to the difference in solvation.

**Fig. 8:**
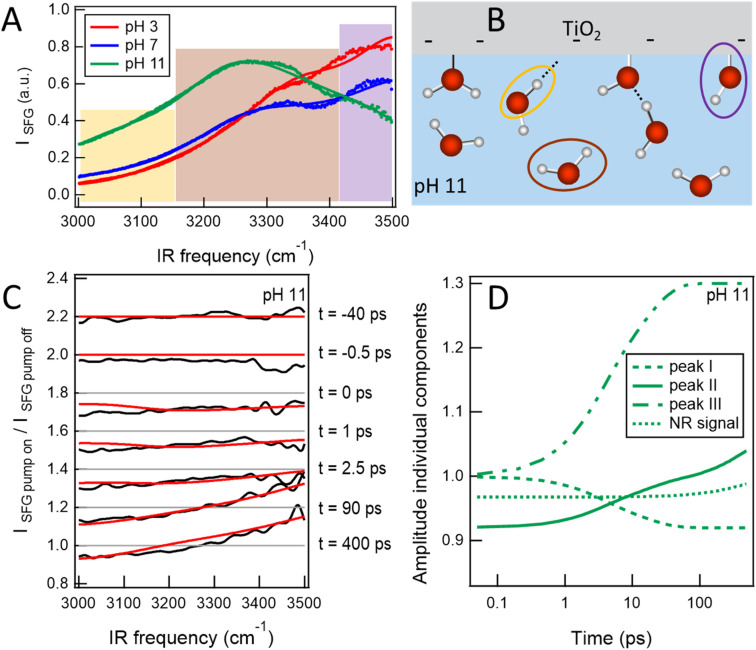
(A) SFG spectra at the TiO_2_–water interface for aqueous solutions of different pH. (B) Schematic of the interface at pH 11 assigning three different water species. The orange, brown, purple circles relate to the low-, central- and high-frequency peaks observed in the spectra of panel A. (C) SFG ratio spectra at several delay times after exciting with 310 nm light. The experimental data are shown in black, while the red lines are results of a model. The model consists of three Lorentzian lineshapes and a NR signal of which the amplitude varies with pump–probe delay time. (D) Time-dependent variations of the amplitude of the individual SFG features. Graphic adapted from ref. 75 under the CC-BY 4.0 license agreement.

Recently, the first reaction at the solid–liquid interface has been followed using time-resolved SFG spectroscopy. Backus *et al.*^75^ studied the dissociation of water at the TiO_2_–water interface after exciting the TiO_2_ with 310 nm light. In this work, an amorphous layer of TiO_2_ (75 nm thickness) was deposited on a 2 mm CaF_2_ plate. By bringing the TiO_2_ film into contact with water, an SFG signal reporting on different water populations was observed, as can be seen in Fig. 8A. For three aqueous solutions with different pH, several peaks in the spectrum could be assigned. For pH 11, the relevant O–H species are depicted in Fig. 8B. The bands centred around 3040, 3220 and 3570 cm^−1^ are assigned to: (i) water molecules strongly bound with their hydrogen atoms oriented towards the interfacial oxygen, (ii) water molecules below the negatively charged surface, and (iii) Ti–OH groups, respectively marked with orange, brown, and purple in Fig. 8B.

In the time-resolved SFG experiment, the SFG probe pulses impinge onto the TiO_2_/water interface from the solid side, as the bulk water would absorb the IR light. The UV light, on the other hand, arrives at the interface from the water side, as the absorbance of the TiO_2_ film would be too high.

Here, we focus on the time-resolved results for pH 11. Upon exciting the TiO_2_ with 310 nm light, the ratio between the pump-on and pump-off spectra show a clear signal above 1 at high frequency, at the frequency of the Ti–OH group (see Fig. 8C), which increases with the delay time between the pump and probe pair. This indicates that Ti–OH groups have been formed and thus that water has dissociated as a result of the photoexcitation of the semiconductor. An analysis of this result is shown in Fig. 8D, which summarizes the intensity of the three different SFG peaks as a function of delay time. It shows that the Ti–OH band (peak III) increases by 30% on a sub-20 ps timescale.^75^

Having succeeded with simple semiconductor systems, the next challenge could be more complex multicomponent catalysts and reactions. An interesting direction might be to also combine sub-ps time-resolved SFG with high-spatial-resolution SFG provided by microscopy.

### 4.5 Sum-frequency generation scattering

SFG has also been expanded to the scattering geometry, making it possible to investigate micron- and nanometre-sized particles that constitute suspensions, emulsions and aerosols.^83–85^ In sum-frequency scattering (SFS), the sum-frequency light is collected at a scattering angle that can be described by a combination of SFG and Mie theory,^86^ with the latter providing a general framework to describe scattering of light by particles in this size range.

SFS has been used to, *e.g.*, reveal the origin of the net negative charge of the interface around hexadecane droplets dispersed in water.^87^ Previous theories have suggested that the negative zeta potential of these hydrophobic interfaces may be attributed to the local accumulation of negatively charged hydroxide ions.^88,89^ However, this was found not to be consistent with the available spectroscopic data.^89^ The authors could show that the origin of the net charge is in fact the formation of improper^90^ C–H⋯O hydrogen bonds. The cumulative effect of this shift in electron density in turn yields the observed net negative charge of the entire droplet. The same technique was also applied to aqueous aerosols,^85^ where the authors made use of both SFS and hyper Raman scattering to simultaneously characterise the bulk and surface composition of aerosol particles containing short-chain organic acids. This combined approach holds interesting promise for the *in situ* analysis of aerosols in different environments and in different size regimes.

## 5 Developments and outlook

The previous sections demonstrated how the chemical and structural information available from vibrational spectroscopy has pushed the frontiers of interfacial science. However, one major disadvantage of traditional spectroscopy techniques is that they are lacking the spatial resolution necessary to describe the structural inhomogeneity of many interfaces. For example, most biological systems and functional materials are inherently structured from the millimetre all the way to the nanometre scale. A complete understanding of these interfaces and their properties can, thus, only be achieved when considering spatial inhomogeneity.

Consequently, there have been efforts to expand spectroscopic techniques to include spatial resolution. Raman and IR spectroscopy were successfully included in microscopy systems to map vibrational spectra with micrometre resolution.^35,91^ Similarly, SFG microscopy has emerged over the last two decades.^92–94^ Compared to Raman and IR microscopy, it has the advantage of being inherently sensitive to molecular orientation, which allows deeper structural insights. SFG microscopy was applied in an exemplary manner to reveal the ordering in lipid monolayers,^92^ map the orientation of methyl groups in a lipid monolayer by changing the azimuthal angle of the sample^95^ and evaluate tissue samples.^96^

While these microscopy-based spectroscopic techniques already brought a significant increase in spatial resolution, they are still inherently limited to the micrometre scale by the diffraction limit of light. To achieve resolution on the nanometre scale, spectroscopy techniques were combined with scanning probe microscopy (SPM). One example is TERS, as discussed in Section 3.1.

**Fig. 9:**
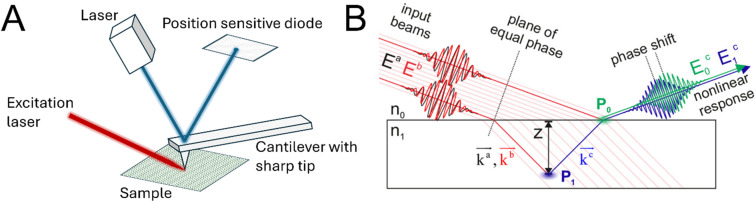
(A) Sketch of the instrumentation for photo-induced force microscopy. The sample is irradiated with a (modulated) tuneable laser, while the scanning probe measures the induced forces. (B) Sum- and difference-frequency signals generated at different probing depths acquire a phase shift as a result of different optical pathlengths. *P*_0_ and *P*_1_ are different radiation sources. Graphic taken from ref. 102 under the CC-BY 4.0 license agreement.

In another effort to expand resolution towards the nanoscale, photo-induced force microscopy (PiFM) was developed in 2010.^97^ Fundamentally, PiFM combines an atomic force microscope (AFM) with an excitation laser impinging on the sample from the side or bottom, as sketched in Fig. 9A. The atomic force microscope is operated in non-contact/tapping mode.^98,99^ To obtain a localised vibrational spectrum of a sample, the photo-induced force is measured as a function of the infrared excitation frequency, where the force modulation changes when the laser coincides with vibrational modes of the sample.^99,100^ The dominant light-induced forces are different depending on the measured sample, *e.g.*, the generally weak dipole–dipole interactions become significant for strongly polarisable objects, while for samples thicker than 100 nm thermal expansion can govern the response.^99^ The non-invasive nature and high spatial resolution makes PiFM especially promising for complex biological samples like bacterial cell sections^98^ or materials science.^101^

With IR and Raman methodologies being combined with scanning probe microscopes, it was just a question of time until nanoscale-resolution SFG spectroscopy was first reported. A report in 2025 introduced scanning tunnelling microscopy with a gold tip being combined with conventional SFG spectroscopy to create tip-enhanced SFG nanospectroscopy.^103,104^ Using this technique, it has been possible to resolve rectangular domains spanning tens of nanometres in a self-assembled monolayer on a gold surface, and to determine domain-specific adsorption structures including up/down molecular orientation.

With SPM combination methods, high spatial resolution along the sample surface/interface is possible, but many interfaces are boundary layers that display more gradual changes between the two bulk phases. For example, electric double layer formation at a charged interface can lead to non-bulk behaviour of water molecules and ions over several nanometres away from the interface. There are already different approaches in SFG spectroscopy to deconvolute the measured spectra at charged surfaces into the Stern and diffuse layer contributions,^105–107^ yet true depth resolution into the molecular organisation across the boundary region stayed elusive. This issue was addressed with the implementation of combined phase-sensitive vibrational sum- and difference-frequency generation (SFG/DFG) spectroscopy in 2022.^102^ In this technique, both the phase-resolved sum- and difference-frequency signal are measured under the same experimental conditions.^108,109^ Generally, a phase difference for signals generated at a different depth is expected due to the different optical pathlength, as depicted in Fig. 9B. This change in pathlength leads to a relative phase shift, which depends both on the depth and the wavevector mismatch. Since the wavevector mismatch of the SFG and DFG process has an opposing sign, the phase difference between the obtained SFG and DFG signal can be used to extract the depth of the signal generation.^108,109^ The principles and underlying theory of sum- and difference-frequency generation spectroscopy were demonstrated on the bulk signal of z-cut quartz and octadecyltrichlorosilane monolayers on fused silica and quartz.^102^ In a later report, the technique was utilised to experimentally determine the depth of the structural anisotropy at the air–water interface. The length found was *ca.* 0.6–0.8 nm, which is in excellent agreement with previous results from simulations.^110^

## 6 Conclusion

Investigating vibrational modes and their associated information about molecular bonding, functional groups and chemical environments has proven itself as an invaluable tool for interfacial research. Experimentally, both linear and non-linear spectroscopic techniques are applied to extract surface-specific information. Infrared reflection–absorption spectroscopy can measure vibrations of (sub-)monolayers of adsorbed molecules at surfaces. Recent new developments enabled the efficient measurement of monolayer adsorbates on even non-metal surfaces. Furthermore, in IR spectroscopy enhancement effects based on surface plasmons and modulation spectroscopy, especially in DRIFTS, have been reported. Also, in Raman spectroscopy, surface plasmons, especially surface plasmon resonance in nanostructured materials, are widely used to increase the signal to noise ratio. In a different approach, both in Raman and IR spectroscopy, the signal of the solvation shell could be obtained by subtracting bulk signals in an appropriate manner.

Another pillar of vibrational interfacial science is sum-frequency generation spectroscopy. By applying the non-linear optical process of sum-frequency generation, this method can selectively probe molecules in non-centrosymmetric environments and a broken centrosymmetry is an inherent property of an interface. The development of time-resolved SFG spectroscopy allows the study of interfacial reactions down to the sub-ps timescale. Yet, the experiments, especially at solid–liquid interfaces, remain challenging and further developments of the technique would help to make such studies more widespread.

Furthermore, there have been significant developments in moving from averaging the vibrational signals over a large sample area towards spatially resolving vibrations at interfaces. Optical microscopy systems have been implemented for all major vibrational techniques – infrared, Raman and SFG spectroscopy. The spatial resolution of such systems is bound by the diffraction limit. Scanning probe microscopies use a sharp tip to probe samples down to the sub-nanometer level. Thus, techniques like tip-enhanced Raman or SFG spectroscopy and photo-induced force microscopy were introduced to push vibrational spectroscopy into the nano-range.

In conclusion, significant progress in resolving and interpreting vibrations has been made. Yet there are still numerous challenges to be addressed in the field.

## Conflicts of interest

There are no conflicts to declare.

## Data Availability

No primary research results, software or code have been included and no new data were generated or analysed as part of this review.
